# Patterns of Spatial and Temporal Distribution of Humpback Whales at the Southern Limit of the Southeast Pacific Breeding Area

**DOI:** 10.1371/journal.pone.0112627

**Published:** 2014-11-12

**Authors:** Chiara Guidino, Miguel A. Llapapasca, Sebastian Silva, Belen Alcorta, Aldo S. Pacheco

**Affiliations:** 1 University of Technology of Sydney, Sydney, Australia; 2 Oficina de Investigaciones en Depredadores Superiores, Instituto del Mar del Perú, Callao, Peru; 3 Pacifico Adventures-Manejo Integral del Ambiente Marino S.A.C., Los Organos, Peru; 4 Instituto de Ciencias Naturales Alexander von Humboldt, Universidad de Antofagasta, Antofagasta, Chile; University of Shiga Prefecture, Japan

## Abstract

Understanding the patterns of spatial and temporal distribution in threshold habitats of highly migratory and endangered species is important for understanding their habitat requirements and recovery trends. Herein, we present new data about the distribution of humpback whales (*Megaptera novaeangliae*) in neritic waters off the northern coast of Peru: an area that constitutes a transitional path from cold, upwelling waters to warm equatorial waters where the breeding habitat is located. Data was collected during four consecutive austral winter/spring seasons from 2010 to 2013, using whale-watching boats as platforms for research. A total of 1048 whales distributed between 487 groups were sighted. The spatial distribution of humpbacks resembled the characteristic segregation of whale groups according to their size/age class and social context in breeding habitats; mother and calf pairs were present in very shallow waters close to the coast, while dyads, trios or more whales were widely distributed from shallow to moderate depths over the continental shelf break. Sea surface temperatures (range: 18.2–25.9°C) in coastal waters were slightly colder than those closer to the oceanic realm, likely due to the influence of cold upwelled waters from the Humboldt Current system. Our results provide new evidence of the southward extension of the breeding region of humpback whales in the Southeast Pacific. Integrating this information with the knowledge from the rest of the breeding region and foraging grounds would enhance our current understanding of population dynamics and recovery trends of this species.

## Introduction

Several taxa of marine megafauna (e.g., sea turtles, albatrosses, cetaceans) undertake long distance migrations between functionally different habitats types, usually from breeding areas to foraging grounds and vice versa. Oceanographic structures such as thermal fronts are key components driving baleen whales movements and foraging patterns as prey aggregates within and surrounding the fronts [1], [2]. The functional role of thermal fronts in breeding areas is less understood since baleen whales feed little during their breeding season [3]. It is thought that humpback whales (*Megaptera novaeangliae*) may use thermal fronts as an environmental cue indicating the proximity of warm neritic waters during their breeding migration from oceanic waters [4], [5]. However, little is known about the distributional patterns of these whales in such habitats.

Among humpback whale populations, the individuals inhabiting the Southeast Pacific region perform the longest migration (ca., 8000 km), moving from Antarctic and Magellanic feeding grounds to the breeding region in neritic waters from the coast of Ecuador up to Costa Rica, crossing the Equator during the austral winter/spring [6]–[8]. The travelled distance is approximately 3000 to 4000 km longer than that estimated for other migrating humpback whale populations worldwide [9]. This extended migration is explained because humpbacks whales seem to search for warmer habitats to avoid the influence of the cold upwelling waters of the Humboldt Current ecosystem extending from central-southern Chile (∼40°S) to northern Peru (∼4°S) during their breeding migration [10], [11]. The average sea surface temperature along neritic waters of the Humboldt Current system during the austral winter and spring months varies between 14–18°C [10], which is colder than the estimated thermal range i.e., 21.1–28.3°C. This is characteristic of breeding and calving areas for humpback whales worldwide [11].

Humpback whales are thought to breed in calm, warm waters because their blubber layer at birth is thin, allowing energy to be invested in growth and development [12], which ultimately enhances calf development and survival. In breeding/calving regions, mother and calf pairs usually prefer to inhabit calm, shallow waters because this may prevent disturbance by competitive males [13], [14] and potential predation from killer whales [15], [16]. This habitat preference explains the conspicuous distributional pattern observed in breeding/calving regions in which groups of whales segregate according to their size/age composition and social context. Although all individuals are present in neritic waters, mother and calf groups typically occupy shallower waters closer to shore, while groups composed of adult (e.g., competitive groups) and sub-adult individuals are widely distributed between shallow, moderate depths and the continental shelf break. This distributional pattern has been recorded in wintering areas of the Southeast Pacific population off the coast of Ecuador [5], [17] and Colombia [18], resembling the patterns observed in breeding regions elsewhere e.g., Costa Rica [19], [20], Hawaii [21], [22], Brazil [23], Madagascar [24] and eastern Australia [25]. However, the segregated pattern may not be evident in all wintering areas as it may depend on site-specific characteristics related to a suite of abiotic factors and the extent of human disturbance. For example, Cartwright et al. [26] found a non-segregated pattern in mother-calf and adult groups' distribution as the whales were present in similar depth ranges close to a boat harbor at the Au'au Channel, Hawaii.

**Figure 1:**
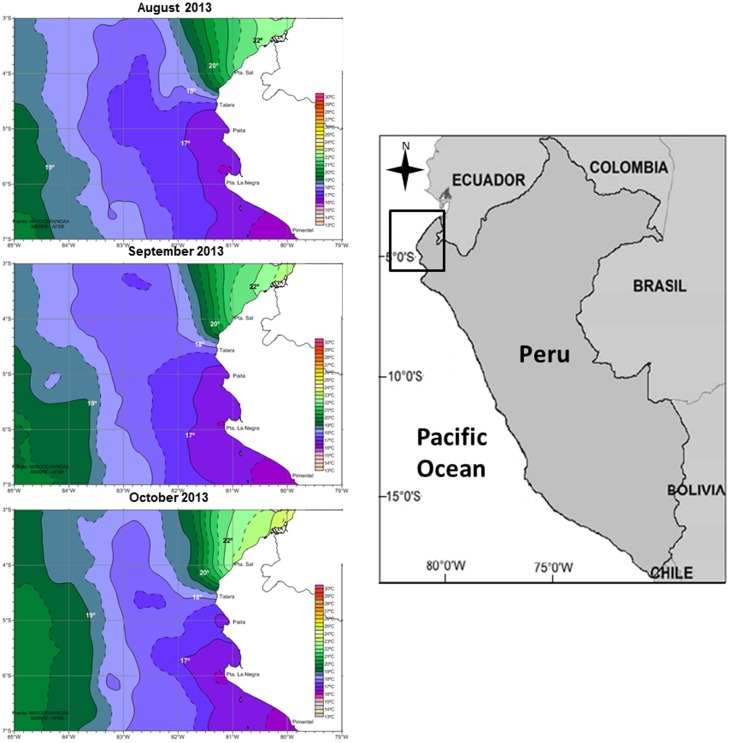
The map at the right shows the location of the northern Peru region. Maps at the left show the distribution of sea surface isotherms (monthly means) during the three months surveys in 2013. The thermal front formed by the convergence of warm equatorial waters coming from the equator and waters of the Humboldt Current system flowing northward is observed. Isotherm images are freely available in http://satelite.imarpe.gob.pe/uprsig/sst_prov.html.

The northern coast of Peru (between 3°–6°S) may constitute a stepping stone during the seasonal migration of humpback whales into the equatorial breeding/calving region, because in this area cold upwelling waters that flow northward of the Humboldt Current ecosystem converge with warm waters coming southwards from the Equator, forming a thermal front (see Fig. 1) that may serve as an indicator for moving from oceanic waters to the neritic breeding/calving region [4], [5], when migrating from high latitudes towards the Equator. However, the available information does not allow clear inferences about the functionality of this region i.e., whether the area serves as a transitional passage during migration or if the region is indeed a functional area for breeding and calving. The assessment of this aspect is important because humpback whales may breed during migration and not only in a defined breeding ground [22]. Peruvian whaling data of the 20th century reports landings of humpback whale adults captured between 80 and 200 miles off the coast in the northern region (∼3°30°S to 8°S) [27], without providing catches or sightings in neritic waters. Humpback whale research since the cessation of whaling has been poor in this region and conducted only opportunistically with very limited spatial and temporal effort, precluding a thorough assessment of habitat functionality [28]. Recently, surveys conducted during individual seasons (from July to November) have reported the presence of mother-calf pairs in shallow waters, very close to the shore along the coast of Los Organos (∼4°S) [29] and Sechura bay (∼5.6°S) [30] (see Fig. 1). In addition, no consistent pattern of directional movement within this region [29] and the conspicuous surface activity throughout the winter/spring season has been reported [31]. Collectively, this information provides new insights about the southern extension of the breeding and calving region in the Southeast Pacific.

Herein, we present new information describing the distribution of humpback whales in neritic waters off northern Peru, encompassing four consecutive breeding seasons (2010–2013). The objective was to assess the functionality of this area for breeding purposes under the prediction that the area is indeed a southward extension of the breeding and calving region. Thus, a segregated distributional pattern according to size/age group will be evident.

## Materials and Methods

### Ethics statement

This study only used noninvasive observational data. No tissues from live or dead whales were collected thus no specific permits were required for the described fieldwork as dictated by Ley Forestal y de Fauna Silvestre del Perú N°29763 (Forestry and Wildlife Law of Peru) and its Reglamento de la Ley Forestal y de Fauna Silvestre through a Decreto Supremo DS No 014-2001-AG (Supreme Decree for regulation). During the course of this study no whales were injured by any means of human interaction (e.g., vessel collision). During surveys, whales were carefully approached following a precautionary set of navigation rules (see details in the boat survey section).

### Study area

We conducted surveys in the coastal area between Los Organos (4°10′38.23″S, 81°8.27′4.83″W) and Cabo Blanco (4°15′1.36″S, 81°13′50.17″W) in northern Peru, during August, September and October from 2010 to 2013. This area is located within a transitional zone between the Tropical Eastern Pacific and the Temperate Southeastern Pacific ecoregions [32]. The transitional zone is made by the convergence between the cold, nutrient-rich Humboldt Current which flows northward and the warm, less productive Equatorial Countercurrent which flows to the East and the South. The coastline in this area is straight without the presence of main inlets or embayments. Surveys were conducted so that neritic waters up to the transition to the oceanic realm over the continental shelf break were covered.

### Boat surveys

**Figure 2:**
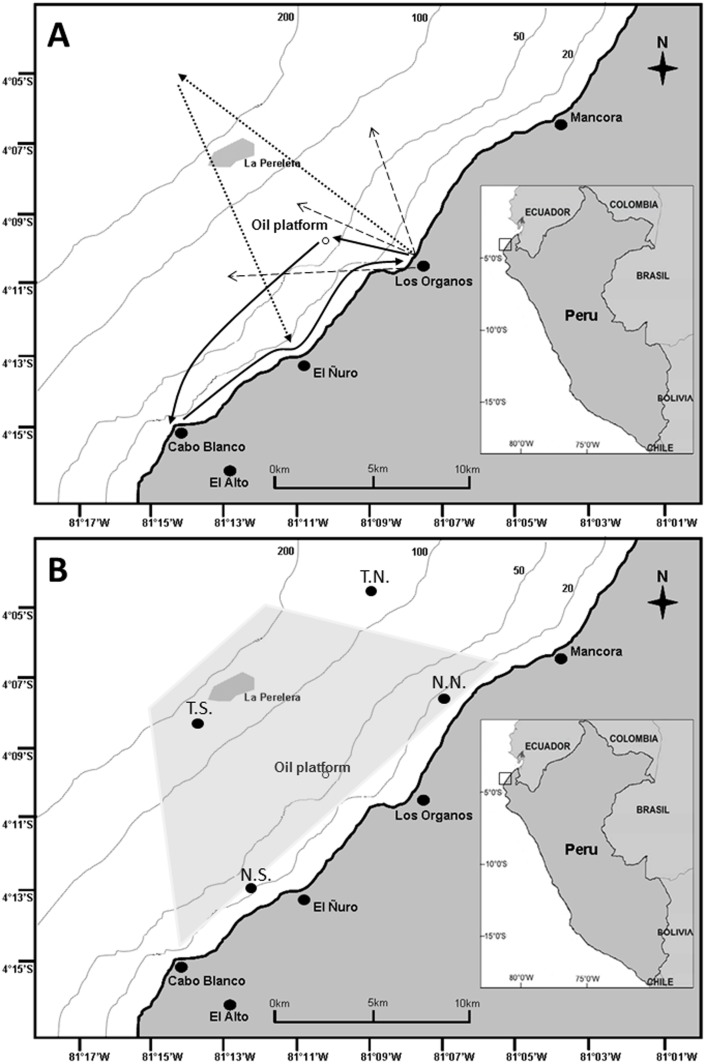
Routes followed during surveys. (A) Solid and dotted arrows are routes during 2010. Dashed arrows are representative of the boats displacement during 2011–2013. (B) Four zones where satellite sea surface temperature values were recorded; neritic north (N.N.), neritic south (N.S.), transitional north (T.N.), transitional south (T.S.). The grey trapeze represents the survey area covering ca. 168 km^2^. Maps were redrawn from the oficial navigation chart of Peru (Carta Náutica del Perú, Dirección de Hidrografía y Navegación, Marina de Guerra del Perú).

Sightings of humpback whales were conducted from whale-watching platforms of research. During 2010, daily surveys were conducted using a boat of 6.7 m length and 2.4 m width with twin outboard engines (85HP each). Navigation from Los Organos started at 7:30 h and took one of two main routes. The first route consisted of sailing to an oil platform as a navigational reference point, heading south to Cabo Blanco, finally returning to Los Organos navigating parallel to the coastline (Fig. 2A). The second route headed to La Perelera bank and further offshore to the north-westernmost point at 14 km, and then returning inbound to El Ñuro and finally back to Los Organos (Fig. 2A). Navigation usually finished at 11.00. In 2011, 2012 and 2013 two more boats (7.9 m length and 2.3 m width with a 150 HP engine and 8.8 m length and 3 m width with twin 200 HP engines, respectively) were added to the sightseeing effort. During these years humpback whales were located by one or two persons sighting whales from the top of a 30 m rocky cliff with the aid of binoculars and directing boat skippers towards the position of the groups via radio communication. The start and end times of the navigation were the same as in 2010. We followed a precautionary set of whale-watching rules during the surveys [33] in order to minimize the potential effect of the whale-watching boats on whales' behavior. Once a whale or group was located, they were approached by skippers that maintained a distance of 30 to 100 m while traveling in the same direction at the same speed as the whale(s). If a whale surfaced close to the boat (i.e. within 30 m), the engine was put in neutral gear until the animal moved away from the boat. Observation time ranged from 10 to 40 min. Observation time for mother-calf pairs were less than 25 min. During surveys, information about the number of whales, relative age/sex class and geographic position (GPS with WGS 84 system) at the closest position of the skipper closest to whales was taken. The depths of the sighted groups were derived from the recorded GPS positions, plotted in a bathymetric map of the area using the application ArcMap in ArcGIS version 10. A group was defined as the total number of animals within ca. 100 m radius, moving in the same direction and usually exhibiting similar displacement/breathing pattern. Occasionally, groups of whales were sighted at close range, but they were not included as part of the group unless they showed obvious interaction with the first sighted group. Even though we used fixed routes during 2010, our data set may be biased because during 2011, 2012 and 2013 surveys were directed by observers on land. At the beginning of the trip, the observers guided the boats to the first visible whale group. After the first sighting boats navigated the area randomly searching for more humpback whales. Thus, the survey effort was never concentrated in a particular location or time. When two or three groups of whales were visible to the land observer, boats were directed to each group individually. In this way, all visible groups were recorded by the boat crew.

### Group composition

The size and composition of the group was determined *in situ* using the following criteria; single, dyad, trio or groups of more than three individuals which generally consisted of relatively large animals (likely adult and/or subadult or a combination of both) following a fairly synchronized breathing and swimming pattern or engaging in competitive behavior (i.e., intense surface activity involving repetitive breaching or whales charging each other, *sensu* [31]). Groups involving the presence of calves include mother-calf pairs, consisting of a fully grown female and its calf, which is an individual with light grey body coloration, measuring at half (or less) the length of a large individual always swimming together. A second type of group arises when the mother-calf pairs are accompanied by an escort: is an escort an individual of equivalent or smaller size than the mother. The final group type is simply a mother-calf pair accompanied by more than one escort.

### Sea surface temperatures

To characterize the thermal variability in the study area, satellite sea-surface temperature data (MODIS-Aqua satellite) was obtained from IMARPE (Instituto del Mar del Peru/Peruvian Sea Institute), which is provided by the National Oceanic and Atmospheric Administration (NOAA) and the NAVOCEANO agency of the U.S. Navy. The information consisted of average daily sea surface temperature taken from August to October during 2010, 2011, 2012 and 2013 at two sampling sites located in the northern part of the study area over a neritic and a transitional location (i.e. from neritic to oceanic waters), and two additional sites located in the southern area in similar waters (Fig. 2B).

### Statistical analysis

Temporal patterns of variation of the group types were studied using simple linear regression with time as a predictor and the abundance of groups (expressed as the number of sighted groups per number of trips during the respective survey month) as the dependent variable. This variable was used in order to reduce the variation due to the differences in sampling effort, i.e., different number of trips in each month. To assess the existence of a segregate pattern in the spatial distribution of humpback whale group types, we followed the approach of Cartwright et al. [26]. We used Neu test [34]–[35] to evaluate the degree of preference for a given bathymetric range (i.e., 0–20, 20–50, 50–100, and 100–200 m depth) considering such ranges as proxies of habitat use. First, the area between two isobaths (in km^2^) in each bathymetric range was estimated and Chi-square test was used to assess the preference of each group type for a given area. Then, Bonferroni ranks were used to estimate whether bathymetric ranges were disproportionally used by humpback whale groups (see [26]). These ranks are confidence intervals of the “Z” Bonferroni's formula which includes the proportion of observed groups and the values of “Z” distribution. The proportions of the expected groups are estimated as function of the area for each bathymetric range. Then, habitat use was designated in a function of the relationship between the expected and observed proportion of the groups and the 95% confidence interval according to the following criteria: (a) avoided; if of the observed proportion of groups in each bathymetric rage was entirely below the expected proportion of groups. (b) preferred, if the observed proportion of groups in each bathymetric rage was entirely above the expected proportion of groups or (c) neutral if the observed groups were contained in the expected proportion of groups within the confidence intervals. Neu's standardized selection indices were also calculated since these provide comparable indices of habitat use. Higher values were assumed to be stronger indicator of preference for a given habitat. Neu's analyses were conducted using pooled data (all months and years) of sighting positions since such analysis need many observations to produce meaningful statistics. Mother-calf pairs with more than one escort group were excluded from this analysis due to the low number of observations. All statistical analyses were performed using the software Statistica 6.0 and PAST version 2.17.

## Results

A total of 279 trips were conducted during the four winter-spring seasons between 2010 and 2013. A total of 1064 whales distributed between 487 groups were recorded in a total of 322.3 hours of observation (see Table S1 for details of groups sighting positions). The most abundant group type was mother-calf pairs (n = 127) followed by dyads (n = 122) and singles (n = 118). Of the total number of groups composed by three or more whales (n = 53), 28.5% (n = 15) were competitive groups. It should be noted that there was some variation in the observational effort between years for example, during 2012 and 2013 the number of trips were twofold compared to the breeding season in 2010 and 2011 (Table 1).

**Table 1:** Summary of the sighting effort and total number of groups per year registered during the study period.

	N°- Trips	Hrs. Observation	N°-Whales (Total)	Single	Dyad	Three or more	Mother-calf	Mother-calf/escort	Mother-calf/>escort
**2010**	71	65.9	241	29	23	5	33	19	4
**2011**	49	51.3	163	19	23	6	20	8	3
**2012**	76	84.1	273	15	43	18	30	10	3
**2013**	83	120.9	387	55	33	24	44	18	2
**Total**	279	322.2	1064	118	122	53	127	55	12

### Temporal variation of whale group types

**Figure 3:**
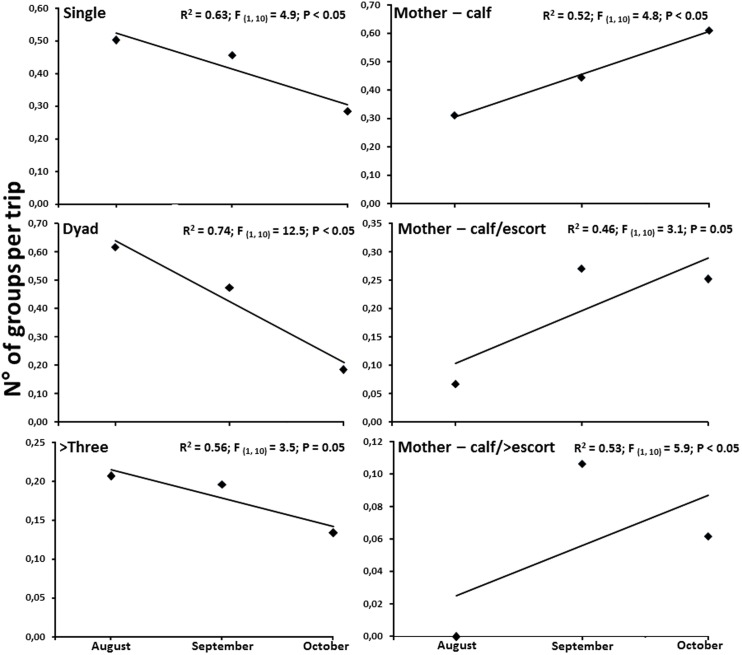
Humpback whales group types. Monthly means (all years) of the number of groups per trip per each sampling month.

Two temporal patterns were clear; singles, dyads and groups made by three or more individuals were more abundant during the first month of the season but tended to significantly decrease towards the end of the study period (Fig. 3). An opposite temporal pattern was evident for mother-calf pairs, mother-calf and escort and mother-calf and more escorts groups, which showed a trend to increase in abundance during the second half of the season (Fig. 3).

### Spatial distribution and habitat preference

**Figure 4:**
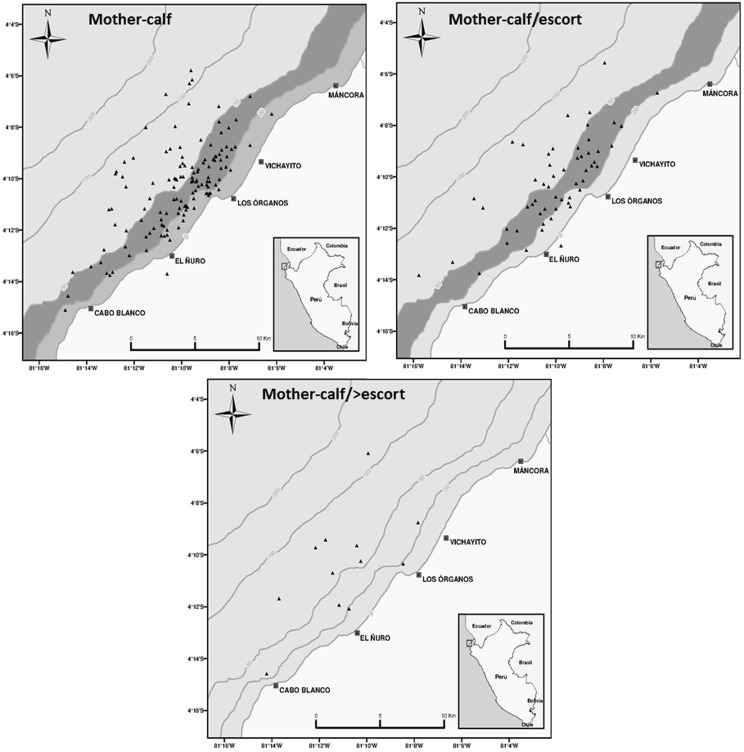
Maps showing GPS-positioned sightings of humpback whales groups; mother-calf pairs, mother-calf and escort and mother-calf and more than one escort during the study period. Most preferred (dark grey) and preferred (light grey) bathymetric ranges are marked after Neu's habitat index.

**Figure 5:**
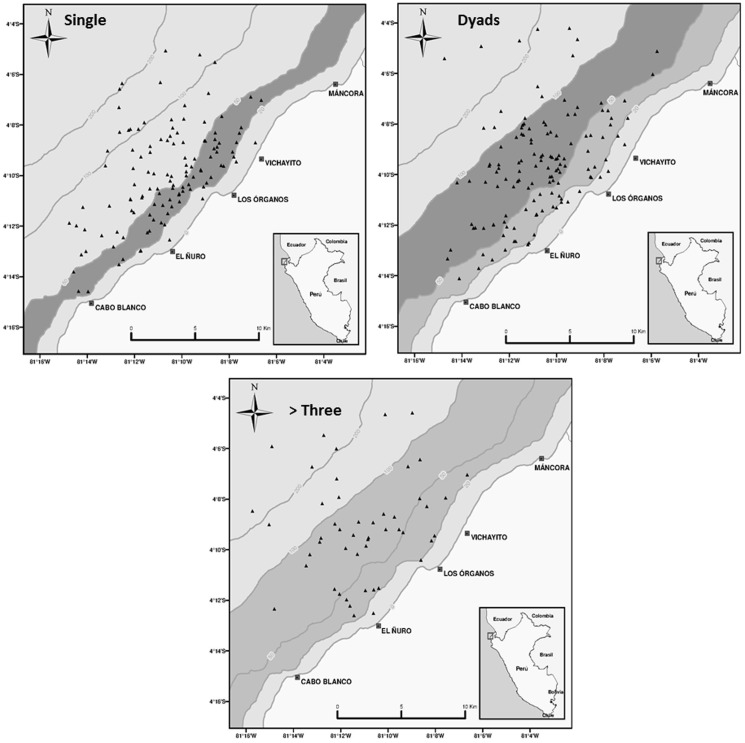
Maps showing GPS-positioned sightings of single, dyads, three or more individuals of humpback whales group during the study period. Most preferred (dark grey) and preferred (light grey) bathymetric ranges are marked after Neu's habitat index.

Of the sightings, ninety eight percent were distributed in the neritic zone *i.e.*, from the shore to the 200 m isobath, but whales density was specific to depth ranges depending on group composition (Table 2). According to the Neu test, mother-calf pairs, and mother-calf and escort groups showed a strong preference for the 20–50 m depth range (Table 3, Fig. 4), although mother-calf groups also occurred in the 0–20 m range. No test was conducted for mother-calf and more than one escort groups but the sighting positions suggest a wide distribution over the survey area (Fig. 4). Singles preferred the 20–50 m range while dyads showed a preference for the 20–50 and 50–100 m depth ranges although a strong preference for the latter range was detected (Fig. 5). Groups composed by three or more individuals showed equal preference for 20–50 and 50–100 m depth ranges (Fig. 5). Overall, a segregated spatial pattern was evident; group of whales with calves, particularly mother-calf pairs preferred shallow areas while groups with no calves were more widely distributed throughout the habitat range.

**Table 2:** Percentage and total number of groups in each depth range registered during the study period.

	Single		Dyad		Three or more		Mother-calf		Mother-calf/escort		Mother-calf/>escort	
Depth (m)	%	n	%	n	%	n	%	n	%	n	%	n
**0–20**	8	10	8	10	4	2	20	25	16	9	17	2
**20–50**	41	48	23	28	23	12	50	64	49	27	25	3
**50–100**	42	49	59	72	51	27	28	35	35	19	50	6
**100–200**	9	11	19	12	23	12	2	3	-	-	8	1

**Table 3:** Summary of Neu's test statistics for humpback whales groups' habitat preference.

	Habitat Depth ranges	Area (km^2^)	Expected groups	Observed groups	Observed proportions	95% C.I.	Proportion of total study area	Neu's Index	Inference
**Mother-calf**	0–20	43.46	18.3	25	0.197	(0.155–0.238)	0.144	0.282	Preferred
	20–50	56.23	23.7	64	0.504	(0.452–0.556)	0.186	0.558	Preferred*
	50–100	121.39	51.1	35	0.276	(0.229–0.322)	0.402	0.141	Avoided
	100–200	80.69	34	3	0.024	(0.008–0.040)	0.267	0.018	Avoided
**Mother-calf/escort**	0–20	43.46	7.9	9	0.164	(0125–0.202)	0.144	0.245	Neutral
	20–50	56.23	10.2	27	0.491	(0.439–0.543)	0.186	0.569	Preferred*
	50–100	121.39	22.1	19	0.345	(0.296–0.395)	0.402	0.185	Avoided
	100–200	80.69	14.7	0	0	0	0.267	0	Avoided*
**Single**	0–20	43.46	16.8	10	0.086	(0.057–0.116)	0.144	0.144	Avoided
	20–50	56.23	21.8	47	0.405	(0.354–0.457)	0.186	0.525	Preferred*
	50–100	121.39	47.1	49	0.422	(0.371–0.474)	0.402	0.253	Neutral
	100–200	80.69	31.3	10	0.086	(0.057–0.116)	0.267	0.078	Avoided
**Dyad**	0–20	43.46	18.7	10	0.0787	(0.051–0.107)	0.144	0.150	Avoided
	20–50	56.23	24.2	32	0.2520	(0.207–0.297)	0.186	0.370	Preferred
	50–100	121.39	52.3	76	0.5984	(0.547–0.650)	0.402	0.407	Preferred*
	100–200	80.69	34.8	9	0.0709	(0.044–0.098)	0.267	0.073	Avoided
**>Three**	0–20	43.46	43.4	2	0.0426	(0.021–0.064)	0.144	0.082	Avoided
	20–50	56.23	56.2	12	0.2553	(0.210–0.301)	0.186	0.381	Preferred
	50–100	121.39	20.9	26	0.5532	(0.501–0.605)	0.402	0.382	Preferred
	100–200	80.69	13.9	7	0.1489	(0.112–0.186)	0.267	0.155	Avoided

“*” denotes higher index values thus regarded as strong preference.

### Sea surface temperature variability

The mean value of sea surface temperature for all study seasons was 22.09°C, with the 2012 season being the coldest and the 2013 the warmest (Table 4). Temperature values were warmer at northern locations compared to the southern part of the study area (Table 4). The south-neritic area was the coldest of all with a mean value of 21.3°C with 18.25°C as minimum value. The warmest area was the transitional neritic-oceanic zone where the mean was 23°C. The neritic-north and transitional neritic-oceanic areas showed fairly similar sea surface temperature values (Table 4). It is worth noting that the ca. 7°C of difference between maximum and minimum sea surface temperature values may account for the strong thermal variability produced by the convergence of the cold upwelling system and warm equatorial waters in the study region.

**Table 4:** Summary of sea surface temperature values recorded during the study period.

	Mean °C	Max °C	Min°C	N.N. °C	N.S. °C	T.N. °C	T.S. °C
**2010**	22.3±1.4	25.9	19.1	22.7±1.3	21.8±1.5	22.8±1.2	22.1±1.4
**2011**	21.7±1.03	25.4	18.2	21.9±1	21.1±1.1	22.2±0.9	21.6±1.05
**2012**	21.3±0.9	23.8	19.2	21.7±0.8	20.1±0.9	22±0.8	21.4±0.9
**2013**	22.9±1.2	24.6	20	23±1.07	22.3±1.1	23.7±1.02	22.6±1.1

Neritic north (N.N.), neritic south (N.S.), transitional north (T.N.) and transitional south (T.S.).

## Discussion

Our results provide new evidence that the northern coast of Peru may not only represent a migratory route from oceanic waters to the neritic realm during their seasonal breeding migration as suggested in the early literature [4]. Humpback whales in our study area depicted the segregate pattern of spatial distribution characteristic of this species in their breeding/calving grounds i.e., groups made up by dyads, trios and more than three individuals were present from moderated depths towards the continental shelf while mother and calf pairs were present in very shallow waters. The record of 194 groups with calves, particularly the presence of mother – calf and escort groups which are conspicuous in wintering regions [36], the presence of large competitive groups adds further evidence of the breeding and calving function of the northern coast of Peru.

The results of this study complement previous information pointing to the breeding functionality of this area such as the absence of a consistent direction in the displacement of the whales suggesting constant movement within the area (e.g., males actively moving searching for receptive females) [29]. The high abundance of single, dyad and mother and calf groups during August, September and October during four consecutive seasons corroborate the functionality of this area as a breeding and calving habitat. Our data shows that groups without calves progressively decrease towards the second half of the season and instead groups with calves increase as the seasons ends. These suggest that although mating activity occurs during the whole season, breeding and calving seems to be more important during the second half of the season [20]. This type of temporal change has been observed in breeding areas elsewhere e.g., Abrolhos archipelago, Brasil, [37]. Groups of humpback whales, including many calves, usually leave the breeding area at the end of the season. Very young calves and new mothers stay in calm waters until the calves are strong enough to undertake the migration south. Unfortunately, our data does not allow us precise information regarding the type of sex/age composition of the individuals at the beginning of the season. We speculate that these may be principally adult females leaving the area after mating, while males may stay and change their reproductive strategy to searching for post-partum mating chances in mother and calf pairs [38]–[39]. This is supported by the increase of groups involving escorts towards the end of the season. Further studies using molecular techniques are still necessary to understand the sequence of arrival and departures of humpback whales in this region.

Spatially, the 98% of the sighted humpbacks were in the neritic zone (<200 m depth) which is similar to the sighting percentage reported in breeding areas elsewhere e.g., [19], [40]–[43]. The existence of a segregated pattern of distribution is a characteristic of breeding and calving regions and such pattern occurs regardless of the width, slope or the distance of the continental shelf break to the coast. For example, the segregated patterns have been observed in locations with a wide and shallow continental shelf such as the breeding region off Puerto Lopez (Ecuador) [43]. While at Salinas (also Ecuador) [5] and Antogil Bay (Madagascar) [23] humpback whales depicted the segregated pattern even though in these locations the continental shelf is narrow with a steep slope. Behavior and social organization finally determine habitat preference of this species: the need for calm, warm waters for calving away from competitive groups is often reported as the main explanation for the coastal distribution of mother and calf groups [12]–[13]. However, geomorphology and anthropogenic impact in the breeding region also play a role in humpback whales local distribution. Cartwright et al. [26] studying the distribution of humpbacks whales at the Au'au channel (an important breeding area of the Hawaiian islands) found a spatial overlap between mother-calf pairs and adults groups, both distributed over 40–60 m depth. The narrowness of the channel together with disturbance by recreational boats in shallow waters seemed to be the cause of this non-segregated spatial pattern [26]. Our results suggest that there is a spatial overlap among mother-calf pairs, mother-calf and escort and singles since these groups shared a strong preference for the 20–50 m depth range. In our study region (∼7°S and further north), the continental shelf break is located at 5–9 km from the coast [44], thus the shelf is rather narrow and steep which may explain the certain degree of spatial overlap among humpback groups before moving into deeper waters. For this study, the influence of the artisanal fishery boats in this coastal region, particularly when gathering at the ports, has not been considered. As suggested before [26], such a concentration of vessels may prevent whales from coming closer to the shore, especially mother and calf groups.

To reveal the segregated spatial pattern of distribution of humpback whales groups, it was necessary that surveys covered coastal areas as well as waters over the continental shelf break. Such spatial cover was achieved during our trips although not systematically during all sampling seasons. For example, during the 2010 season the first navigation route was fixed and always reached waters over the 200 m isobaths. However, during the 2011–2013 seasons' navigation to deeper waters were less frequent. As we previously stated, such differences in survey effort may bias results since sighting rates of a given group type may differ between random navigation and fixed transect surveys. Despite of this methodological shortcoming, our data revealed significant temporal and spatial patterns of distribution. However, we suggest caution when comparing these results with studies using transect surveys.

The variability of sea surface temperature in our study region revealed that humpback whales experience rather colder conditions; the mean was 21.5°C during all the seasons which is three degrees lower than the average estimate for breeding regions worldwide [11]. Indeed, we registered minimum values of ∼18.5°C in the southern-neritic zone. Migratory humpback whales in the Southern Hemisphere oceans breed at ∼14°S, (although recent surveys demonstrate the existence of breeding areas near the equator in the eastern tropical Atlantic e.g. Gulf of Guinea for some South Atlantic stock [45]) while the southeast Pacific stock extends its migration close to lower latitudes and even crossing the equator [6], [9]. Early literature suggested that humpback whales would avoid the influence of the cold neritic conditions imposed by the Humboldt upwelling ecosystem and humpback whales would approach neritic waters when encountering warm conditions further north at the equatorial realm [4]. However, our results suggest that the northern coast of Peru may constitute a threshold between upwelling conditions and equatorial waters and such transitional habitat may constitute an extension of the breeding and calving region. Sea surface temperature values of this region ranged between the lowest values reported for other breeding regions e.g. 19–20°C at Bonin and Ryukyu Islands, Japan [11]. However, much research is still needed to reveal the influence of the thermal gradients on humpback whales distribution and habitat requirements, particularly for mother-calf pairs and newborn calves. For example, there are two existing observations of humpback whale parturition [46], [47] and only the observation in Brazilian waters reports a sea surface temperature of 24°C at the moment of birth [46]. Although very preliminary, this may suggest that births occur in warmer areas inside the breeding region and that zones with slightly colder temperatures may function principally as habitats for calf development. However, the fact that we recorded calves in relatively colder temperatures indicates that they are capable of surviving these conditions and may not need to be born in warmer waters. Detailed studies describing the morphology and behavior of the calves e.g. [13] along the breeding region are necessary for a better understanding of the dynamics of this delicate life stage during the life of a humpback whale. It is important to mention that the population of humpback whales is increasing [48] and likely their presence in neritic waters off Peru may constitute an indicate of breeding range expansion. It is possible that breeding in more northern areas occurred in the past because these areas were the optimal habitats. Currently, these areas may be saturated and whales are looking for other breeding areas further south. The presence of several calves reported here supports this notion.

While we provide new information about the distribution of humpbacks at the southern limit of the breeding and calving region, there is also renewed evidence of a northward recovery of the former feeding grounds in the Patagonia region [48], [49]. Collectively, these apparent extensions of both breeding and feeding regions suggest that this species is likely recovering and returning to its former distributional ranges before the industrial whaling period. This conclusion is supported with recent estimations of population size, suggesting steady increments throughout time during the last *ca.* 20 years [50]. However, it should be noted that these numbers may be underestimated since calculations were based on data obtained in a fraction of the breeding area (i.e., off Ecuador). Enhancing multinational research efforts along the Southeast Pacific would improve our ecological knowledge and population status of this species which also would improve management and conservation efforts for this species.

### Outlook

In this study, we have highlighted the neritic presence of humpback whales during their breeding migration off the coast of northern Peru. However, this coastal distribution makes the species prone to entanglement and mortality with gillnet fisheries [51]. It is mandatory to regulate the use of such fishing gears during the seasonal presence of this species in neritic habitats. In ecological terms, little is known about the displacement patterns of this species within the breeding region. Satellite tracking and photo-identification research may reveal the timing and specific habitat requirements during the breeding season and these are topics that should be addressed in the near future. Although, we acknowledge the use of whale-watching boats as platforms for investigation opportunities for, humpback whale research in this region should be complemented with other types of sampling such as focal-group followed and transect surveys aimed at testing specific hypotheses on ecological and behavioral aspects of this species.

## Supporting Information

Table S1 **Humpback whales off northern Peru; sighting positions, group composition during 2010–2013 winter/spring seasons.**
(XLSX)
